# Multimodality cardiac imaging and new display options to broaden our understanding of the tricuspid valve

**DOI:** 10.1097/HCO.0000000000000890

**Published:** 2021-07-20

**Authors:** Valentina Volpato, Luigi P. Badano, Stefano Figliozzi, Diana R. Florescu, Gianfranco Parati, Denisa Muraru

**Affiliations:** a Department of Medicine and Surgery, University of Milano-Bicocca; b Department of Cardiac, Neural and Metabolic Sciences – Istituto Auxologico Italiano IRCCS, Milan, Italy; c Department of Cardiology, University of Medicine and Pharmacy of Craiova, Craiova, Romania

**Keywords:** multimodality imaging, tricuspid regurgitation, tricuspid valve

## Abstract

The prognostic impact of tricuspid regurgitation (TR) and the subsequent development of percutaneous procedures targeting the tricuspid valve (TV), has brought to the forefront the role of imaging for the assessment of the forgotten valve. As illustrated in several studies and summarized in this review, currently a multimodality imaging approach is required to understand the pathophysiology of TR, due to the complex TV anatomy and the close relationship between the severity of TR and the extent of the remodeling of the right heart chambers.

**Recent findings:**

Recently, the advance in the tranhscatheter treatment of the TV has led to a growing interest in the development of dedicated software packages and new display modalities to increase our understanding of the TV. As a consequence, a transversal knowledge of the different imaging modalities is required for contemporary cardiac-imaging physicians.

**Summary:**

This review highlights the main features, and the pros and cons of echocardiography, cardiac computed tomography, cardiac magnetic resonance and emerging technologies, as 3D printing and virtual reality, in the assessment of patients with TR.

## INTRODUCTION

Tricuspid regurgitation (TR) is associated with significant morbidity and mortality [1,2]. Current guidelines recommend tricuspid valve (TV) surgery in patients with severe TR and either symptoms or progressive right ventricular (RV) dilation or dysfunction. Additionally, TV repair should be considered in patients with mild-to-moderate TR due to tricuspid annular (TA) dilatation undergoing left-sided valve surgery and in cases with right-sided heart failure and severe isolated TR secondary to TA enlargement [3,4]. However, the high surgical mortality (8–10%) of isolated TV surgery [5], and the several comorbidities characterizing these patients [6^▪^], resulted in a relatively undertreatment of TR [5]. To meet this growing clinical need, several devices have been developed for the percutaneous treatment of TR providing an alternative to surgery in high-risk patients [7–9].

To properly identify patients who need TV repair and correctly address them to percutaneous vs surgical treatment, we need to: i. assess both etiology and severity of TR; ii. quantify the right heart chambers’ dimension and RV function; iii. sizing the TA; iv. estimate the pulmonary artery pressure [10^▪▪^]. Currently, the accurate assessment of the unique TV geometry, and the complex RV shape and mechanics, require a multimodality imaging approach that encompasses the use of three-dimensional echocardiography (3DE), cardiac computed tomography, cardiac magnetic resonance (CMR), as well as new emerging imaging modalities [11,12]. 

**Box 1:**
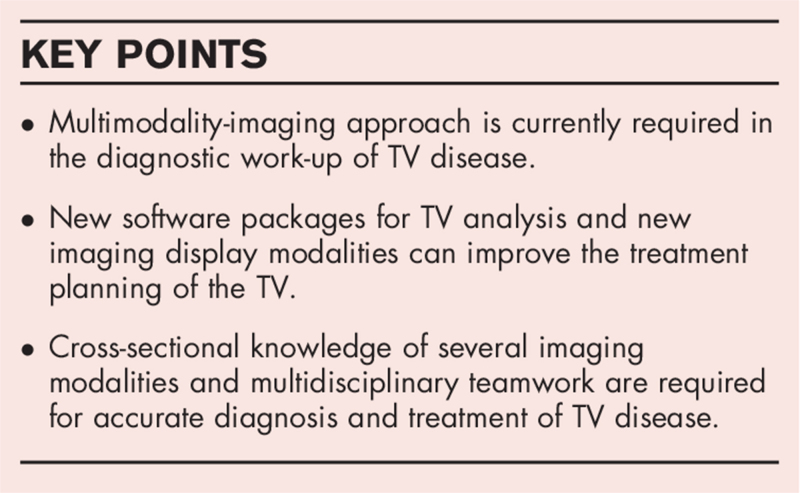
no caption available

## IMAGING OF THE TRICUSPID VALVE

**FIGURE 1:**
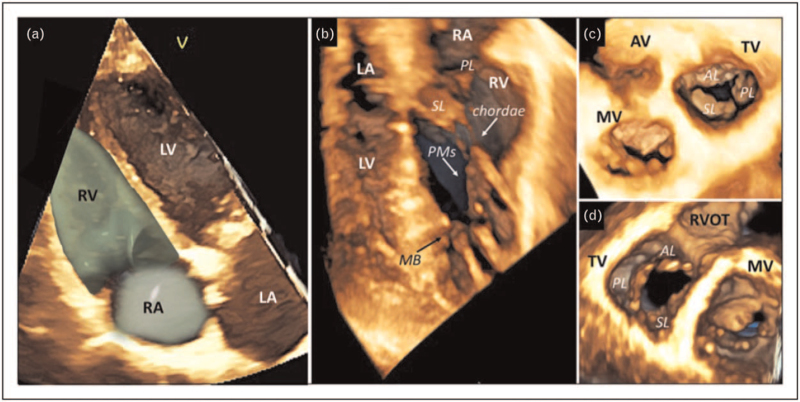
Three-dimensional assessment of the right ventricle and right atrium using transthoracic echocardiography (a). Evaluation of the subvalvular apparatus using transthoracic three-dimensional dataset showing tricuspid valve leaflets, chordae, papillary muscles and the moderator band (b). Three-dimensional echocardiographic display of the tricuspid valve form the atrial perspective (c) and ventricular perspective (d). AL, anterior leaflet; AV, aortic valve; LA, left atrium; LV, left ventricle; MB, moderator band; MV, mitral valve; PL, posterior leaflet; PMs, papillary muscles; RA, right atrium; RV, right ventricle; RVOT, right ventricular outflow tract; SL, septal leaflet; TV, tricuspid valve.

The complex TV apparatus relies on several structures acting in a concerted way in a low-pressure system. These structures are crucial in valve continence and are functionally related to RA and RV geometry and function (Fig. 1).

### Tricuspid valve leaflets

The TV is a 3-leaflet valve, even if the number of leaflets may vary [13]. The posterior leaflet might present multiple scallops and in up to the 10% of the population the anterior and posterior leaflets are merged [14^▪▪^]. The anterior leaflet is the largest in both circumferential and radial directions, the posterior has the shortest circumferential extension whereas the septal has the shortest radial extension. TV leaflets are thinner than the mitral ones and calcification rarely occurs. The three commissures, antero-septal, antero-posterior and postero-septal are defined by the free edges of each leaflet and a fan-like chorda is usually present [15].

**FIGURE 2:**
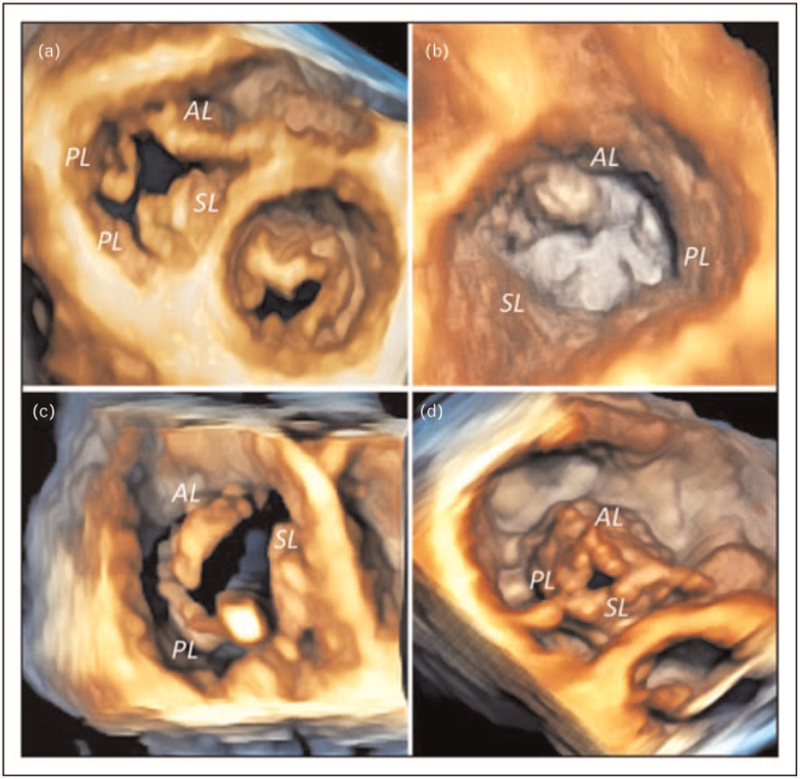
A quadricuspid tricuspid valve showing the presence of two distinc posterior leaflets (a). Prolapse of the anterior leaflet of the tricuspid valve (b). Pacemaker lead impingement on the posterior leaflet in a patient with tricuspid regirgitation (c). Coapation gap defect at end systole in a patient with severe tricuspid regurgitation (d). AL, anterior leaflet; PL, posterior leaflet; SL, septal leaflet.

**FIGURE 3:**
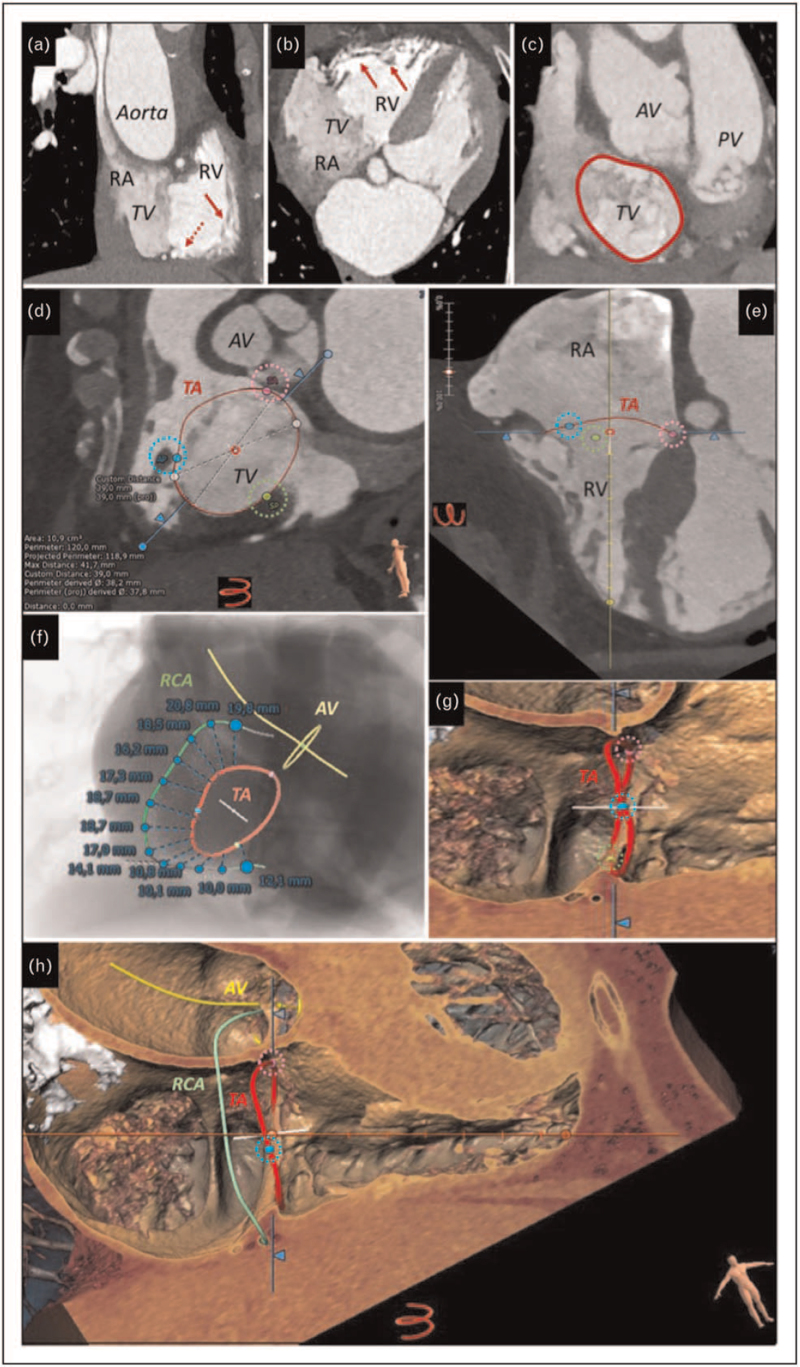
Cardiac computed tomography assessment of the tricuspid valve, right ventricle, chordae (dashed red arrow) and subvalvular apparatus (red arrows), using an inflow-outflow right ventricular view (a) and a 4-chamber view (b). Tomographic reconstruction of the tricuspid annulus with measurement of annular perimeter (c; courtesy of Dr Giuseppe Muscogiuri, Centro Cardiologico Monzino IRCCS, Milan, Italy). Dedicated software analysis providing three-dimensional reconstruction of the tricuspid annulus (red) and measurement of circumference, area and diameters using cardiac computed tomography datasets (d, e; courtesy of Pie Medical Imaging, The Netherlands); identification of commissures is provided by the software (septal-anterior commissure, dashed pink circle; antero-posterior commissure, light blue dashed circle; septal-posterior commissure, green dashed circle). Localization of surrounding structure by dedicated tricuspid valve software using computed tomography derived virtual angio, providing localization of the aortic valve and identification of the right coronary artery; distance between the tricuspid annulus and the right coronary artery is automatically measured by the software (f; courtesy of Pie Medical Imaging, The Netherlands). A zoomed three-dimensional cardiac computed tomography display of the right atrio-ventricular junction showing the three-dimensional reconstruction of the tricuspid annulus and identification of commissures (g; courtesy of Pie Medical Imaging, The Netherlands). Three-dimensional cardiac computed tomography rendering of the right heart with three-dimensional reconstruction of the tricuspid annulus, identification of commissures and anatomical relationship with the aortic valve and right coronary artery (h; courtesy of Pie Medical Imaging, The Netherlands). AV, aortic valve; PV, pulmonary valve; RA, right atrium; RCA, right coronary artery; RV, right ventricle; TA, tricuspid annulus; TV, tricuspid valve.

As showed by Addetia *et al.*, two-dimensional (2D) transthoracic echocardiography (TTE) has a fair chance to identify TV leaflets using conventional echocardiographic views [16]. Moreover, due to the high variability in spatial orientation of the TV [17], 2D TTE still presents several pitfalls and rarely allows a simultaneous display of the three leaflets that could be obtained only using the transgastric basal view by transesophageal echocardiography (TOE) [18]. Nonetheless, TOE is limited by the position of the TV in the anterior mediastinum (in the far field of probe) and, as for TTE, results in inaccurate identification of leaflets. 3DE overcomes the limitations of 2D echocardiography (2DE), and TTE is the method of choice due to the position of the TV [13,19^▪▪^]. Real-time 3DE acquisition is preferred to avoid stitching artefacts related to inadequate breath-hold or arrhythmias, and its useful during transcatheter procedures [11,20]. When a higher frame-rate is required, particularly using 3DE color Doppler, the multibeat acquisition is desirable [11]. Due to the TV position and orientation, any TTE acoustic window might be used for 3DE dataset acquisition. Conversely, TOE can be more challenging and the mid- and deep-esophageal transgastric views are preferred for image acquisition. 3DE datasets allow a comprehensive assessment of the three leaflets, and the structural abnormalities of the TV can be visualized by the enface atrial or ventricular perspectives (Fig. 2). The development of dedicated semi-automated software for TV assessment can provide a quantitative analysis not only of the annulus, but also of the extent of leaflet tethering [13] (Fig. 3), both associated to severe functional TR (FTR) [21,22^▪▪^,^23^,^24^]. Important 3DE limitations in imaging the TV are either the artefactual excessive thickening of the leaflets or dropouts [25].

Recently, new software packages have been developed for multidetector row computed tomography (MDCT) that are particularly advantageous in preprocedural planning. Dedicated tools are mainly used for TA sizing. However, using end-systolic MDCT data (30–40% of cardiac cycle) they provide also measurement of the extent of leaflet tethering or the 3D tenting volume, as well as landmark structure localization [26–28] (Fig. 3). Kabasawa *et al.* showed that these parameters are useful in predicting the recurrence of TR after surgical annuloplasty [29]. Evaluation of TV and right heart function by electrocardiographic (EKG)-gated MDCT requires dedicated sequences covering the entire cardiac cycle and adequate opacification of the right heart via contrast agent. Increase in *z*-axis coverage allows image acquisition with lower breath-hold time, radiation and contrast agent doses, whereas good temporal resolution can be achieved by the use of dual-energy scanners [30].

**FIGURE 4:**
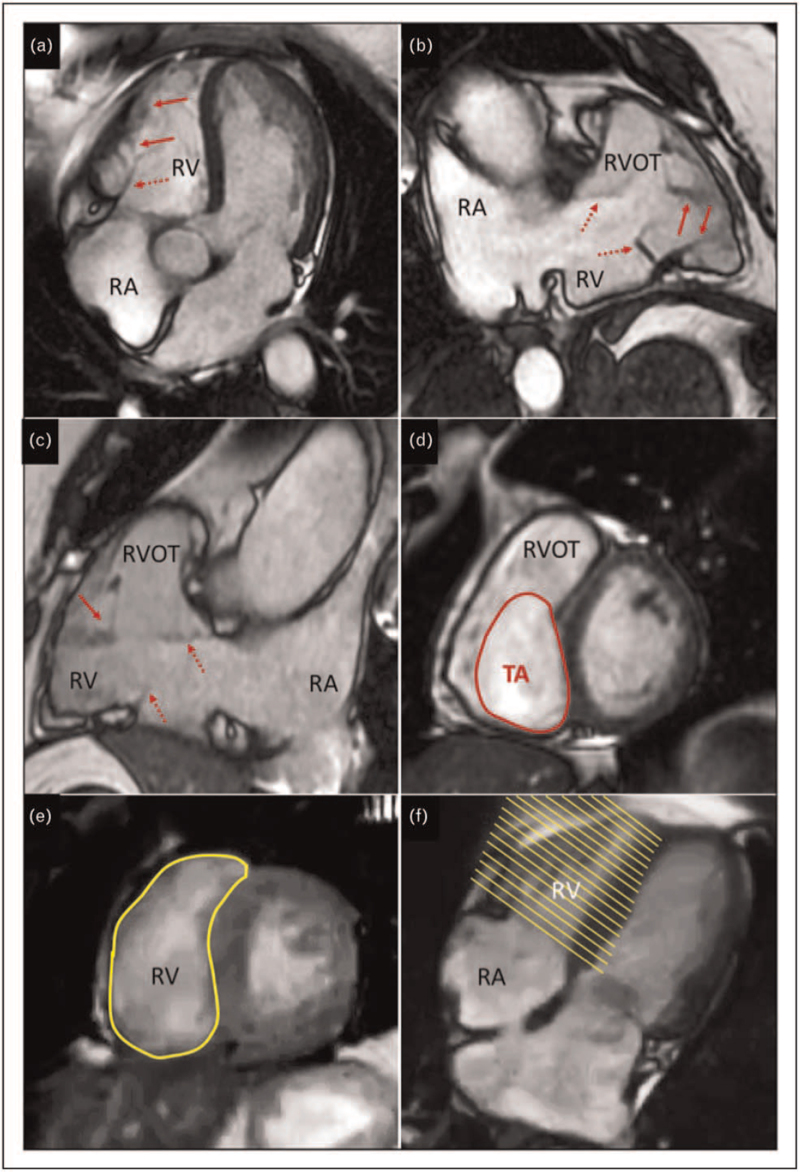
Assessment of the tricuspid valve leaflets (dashed red arrows), chordae and papillary muscles (red arrows) using 4-chamber view (a), right ventricular 2-chamber view (b) and right ventricular inflow-outflow view by cardiac magnetic resonance in a patient with Ebstein disease (c). Tomographic reconstruction of tricuspid annular perimeter using dedicated short axis acquisition (d). Assessment of the right ventricular volume and function by direct tracing of the endocardial volume (e, f). RA, right atrium; RV, right ventricle; RVOT, right ventricular outflow tract; TA, tricuspid annulus.

Leaflets evaluation by EKG-gated CMR using 1.5 or 3.0-T scanners suffers from the lower temporal resolution of CMR compared to echocardiography that limit the visualization of highly mobile structures, as flail scallops. Using standard RV long-axis views (4-chamber, RV inflow-outflow) [31] only two out of three leaflets can be displayed simultaneously and, as for 2DE, leaflets cannot be accurately identified. Abnormalities of TV leaflets can be detected, but dedicated short-axis continuous stacks of thin slices without gap can be necessary albeit an extra scan time is needed [31] (Fig. 4). Arrhythmias represent an important limitation for CMR that can be overcome using free-breathing sequences with acceptable spatial and temporal resolution [32].

### Tricuspid annulus

Rather than being a distinct structure, the TA is a functional entity delimited by muscular fibers from the RA cavity, adipose tissue of the atrioventricular groove, and a little amount of fibrous tissue [15,33,34^▪▪^]. The partition of the TA follows the leaflets, the septal segment is attached to the membranous septum and the right fibrous trigon, explaining why it is usually spared from elongation. The TA presents two higher points, anteroseptal and posterolateral, and two lower points, anterolateral and posteroseptal [35], and its dynamic nature led to a decrease up to 20% in area and 30% in perimeter at late systole compared to late diastole [36].

**FIGURE 5:**
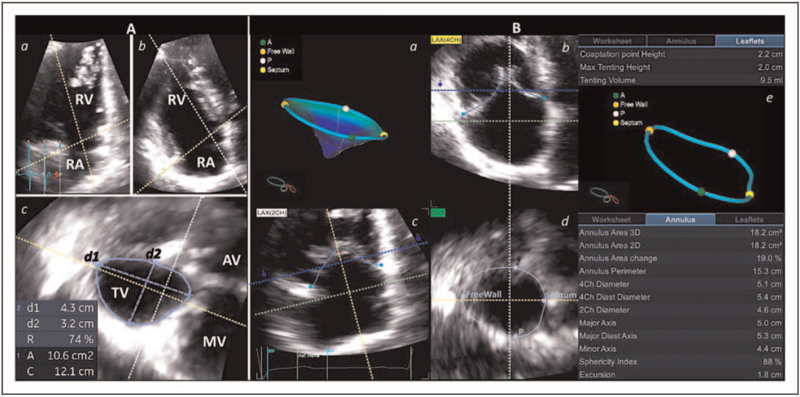
Panel A. Three-dimensional assessment of the tricuspid annulus using multiplanar reconstruction analysis. Orthogonal planes are adjusted at end-diastole in order to cross the tricuspid valve hinge points using an apical right ventricular 2-chamber (a) and 4-chamber (b) views. Measurement of tricuspid annular perimeter, area and diameters are obtained using the axial plane (c). Panel B. Semi-automated analysis of the tricuspid valve using dedicated software (GE Healthcare, Vingmed, Horten, Norway). After initialization of anatomical references by the reader, the software displays the three-dimensional reconstruction of the tricuspid valve (a) and tricuspid annulus (e). The reader can double check and edit using orthogonal planes (b–d). Measurement of tenting height and volume and tricuspid annular circumference, area, diameters and sphericity index are automatically provided.

2DE has been demonstrated to be inaccurate in measuring the TA because its complex shape makes linear measurement intrinsically view-dependent [37]. However, 2D TTE is still recommended for TA sizing because it is easy-to-perform, widely available, safe and cheap [3,4], despite the limited scientific evidence supporting the currently used cut-off value for TA dilatation requiring surgery (40 mm or 21 mm/m^2^) [37,38]. 3DE TA analysis has been proven to be more accurate and reproducible [39]. The multiplanar reconstruction (MPR) analysis represents an easy-to-use method for measuring TA diameters, circumference and area (Fig. 5), even if MPR does not take into account the 3D shape of TA, and the measurements are performed on a projection of the 3D annulus on a 2D surface [35]. This might result in underestimation of actual dimensions compared to tools that account for the 3D annular geometry [39]. Commercially available software packages for TV analysis allow a simultaneous assessment of both leaflets and annulus, providing measurements of TA diameters, perimeter and area, and taking into account the changes of the 3D shape during the cardiac cycle [11,13,19^▪▪^] (Fig. 5).

Nowadays, TA assessment by MDCT sequences is mandatory for procedural planning [40]. As for echocardiography, TA dimension can be assessed using MPR at end-diastolic phase (70–80% of cardiac cycle) or using dedicated automated software packages that take into account the 3D geometry and provide an accurate TA sizing [26–28] (Fig. 3).

Both by echocardiography and MDCT, the TA is identified after indexing the hinge point of the TV leaflets. Conversely, by CMR the TA is defined by the adipose tissue in the atrioventricular groove and its extension up to the TV hinge points [41]. When CMR is used, the tomographic TA reconstruction is performed using whole heart sequences or dedicated short-axis parallel to the TA plane (Fig. 4), and presents the same above-mentioned limitations as for MDCT and echocardiography.

### Subvalvular apparatus

The subvalvular apparatus involves chordae and papillary muscles (PMs) and may present some degree of variability [15]. Primary, secondary and marginal chordae are attached to the base, body and free edges of the TV leaflets, respectively. Chordae arising from the anterior and posterior PMs reaches both the anterior and posterior leaflets, whereas chordae supporting the septal leaflet arise directly from the septum. The anterior PM is double or triple-headed, is the largest and the most apically displaced. Together with the moderator band and the trabecula septo-marginalis divides the RV inflow from the outflow tract. The posterior and septal PMs might have multiple heads or multiple thin muscles arising from the posterior and septal wall, respectively. Dislocation of PMs secondary to RV remodeling has been associated to the development of FTR [15,33].

The PMs or highly mobile structure, as chordae, can be visualized using both 3D TTE and TOE (Fig. 1). Particularly, TOE is helpful in displaying the subvalvular apparatus during percutaneous procedures [14^▪▪^]. However, MDCT is the method of choice for this purpose given the high spatial resolution of the modality. Both the distance from the annulus to the RV apex and distance from the PMs to the annulus and RV septum measured by MDCT, as well as visualization of chordae insertion, are critical information for percutaneous procedures [14^▪▪^,^19^^▪▪^,^28^] (Fig. 3). CMR is not routinely used due to frequent artefacts related to the thin and mobile chordal structure (Fig. 4).

### Right chambers

The RV is crescent-shaped and can be divided in three portions, inlet, apical and outflow. This complex shape makes 2DE unsuitable for RV imaging [15]. Measurement of volumes and function by 3D TTE showed a good correlation with CMR [12,42], that is still the gold standard for RV volume measurement providing also information about tissue characterization. The RA is a composite structure made by an anterior trabeculated portion, anatomically related to the annulus, and a posterior smooth portion in relationship with the septum and the venae cavae [15]. RA quantitation by 2DE relies on monoplane linear and area measurement to calculate the volume using the area-length algorithm and does not take into account the RA geometry that can be accurately assessed using 3DE [42,43]. RA measurement by CMR is routinely performed by area-length method using the 4-chamber view at end-systole, otherwise dedicated short-axis stacks can be used for volumetric computation albeit extra scan time is required [44]. Also, MDCT measurement of RV volume and function demonstrated a good correlation with CMR, making MDCT a good alternative in patients with limited echocardiography window and unsuitable for CMR [45,46], (Figs. 1 and 4).

Both the RV and the RA play a crucial role in FTR development. The dilatation of the RA and/or RV cavity can led to TA enlargement [34^▪▪^,^47^–^52^], and conical RV remodeling can cause TV tenting [53].

### Surrounding structures

Several vital structures have a close anatomical relationship with the TV. The right coronary artery runs in the atrioventricular groove surrounding the TV, the bundle of His and the aortic valve are close to the anteroseptal TA segment [15].

Imaging of adjacent structures by TOE, as the coronary sinus or the venae cavae, helps the interventional cardiologist to identify landmark structures, that cannot be visualized by fluoroscopy [54]. MDCT is the cornerstone in this setting, providing accurate measurement of the distance between the TA and the right coronary artery (necessary for annuloplasty systems or transcatheter TV replacement), and the diameters of the venae cavae and subclavian/axillary arteries required for access planning [55], (Fig. 3).

### Tricuspid regurgitation

Disease affecting any of the above-mentioned structures can cause TR [56], even if FTR is the most common etiology [57^▪▪^,^58^–^61^].

**FIGURE 6:**
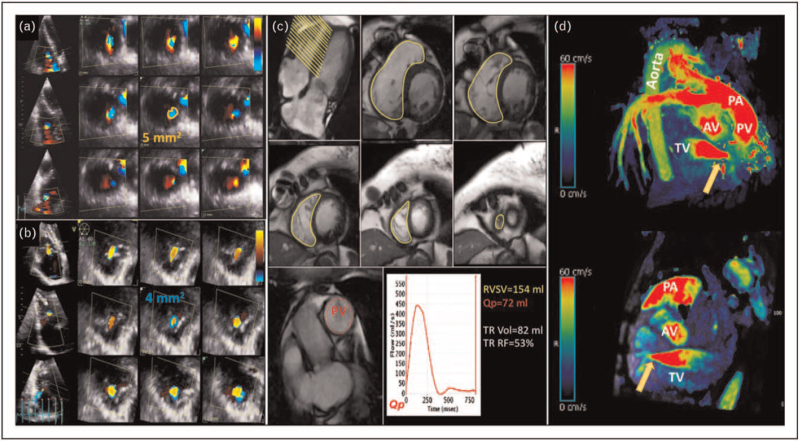
Measurement of three-dimensional vena contracta by transthoracic echocardiography in a patient with functional tricuspid regurgitation (a). Measurement of three-dimensional effective regurgitant orifice area by transthoracic echocardiography in a patient with severe tricuspid regurgitation (b). Quantification of tricuspid regurgitant volume and fraction using cardiac magnetic resonance (c). Regurgitant volume is derived by the difference between the right ventricular stroke volume (yellow) and the forward pulmonary valve stroke volume (red). Assessment of tricuspid regurgitation (orange arrows) using cardiac magnetic resonance 4-D flow images with flow velocity encoded in all directions (d; courtesy of Dr Giuseppe Muscogiuri, Centro Cardiologico Monzino IRCCS, Milan, Italy). PA, pulmonary artery; PV, pulmonary valve; TV, tricuspid valve.

The TV is set in a low-pressure system which makes the severity of TR dependent on loading conditions and respiratory variations. This background in combination with 2DE limitations explains why TR quantification is still a challenging field. Current guidelines recommend a multiparametric assessment of TR [3,4] even if parameters as vena contracta (VC) and effective regurgitant orifice area (EROA) using proximal isovelocity surface area (PISA) method rely on geometrical assumptions that are rarely met in TR [62,63]. Particularly, these parameters postulate that the regurgitant orifice is plane and circular, whereas it can present different morphologies, and changes in size during the cardiac cycle and breathing condition [10^▪▪^]. Indeed, the VC and EROA are widely used for TR quantification and recently new grading for FTR severity have been proposed using these parameters [64–66]. Several studies suggested to measure the irregular VC area using the tomographic 3D reconstruction (Fig. 6). Currently, validated cut-off values for 3D VC area are lacking, and the discrepancies in the proposed thresholds [67–69] could be related to the methods used for area measurement. Minor changes in planes alignment, in color Doppler gain setting or in the frame used can significantly change the resulting area of the VC. The use of 3D PISA could be advantageous in measuring the irregular iso-velocity surface volume of TR (Fig. 6), even if few data are available [70]. However, this method may result in underestimation of the real volume because the color Doppler technique is angle-dependent making volume reliant on probe alignment [57^▪▪^]. Additionally, the formula requires the use of TR systolic peak velocity that is a static parameter, thus preventing EROA measurement throughout the cardiac cycle. The 3D assessment of the regurgitant volume is likewise limited by the TR systolic peak velocity as it depends on the pressure gradient between the RV and RA chamber and does not take into account the lifetime of TR throughout the cardiac cycle [57^▪▪^]. Recently, the calculation of the regurgitant fraction using a combination of 3D and Doppler techniques has showed to have prognostic value and a threshold value of 45% has been proposed to identify high-risk patients [65].

CMR have the advantage to directly calculate TR volume and regurgitant fraction using standard phase-contrast sequences [31,71,72] (Fig. 6). Lately, Zhan *et al.* proposed new cut-off values for TR regurgitant volume and fraction based on their independent prognostic correlation with survival in patients with FTR [73]. Novel 4D-flow velocity-encoded technique can directly measure the regurgitant volume using whole heart free-breathing sequences with velocity encoded in all flow directions (Fig. 6) and seem to be promising for valvular diseases [74,75].

The high spatial and temporal resolution of MDCT allow direct measurement of EROA at end-systolic phase on top of visualization of leaflets and subvalvular apparatus [29]. The computation is performed by MPR analysis and share the same limitations of the tomographic reconstruction by echocardiography and CMR for TR quantification, except for the use color Doppler.

### Fusion, printing and virtual reality

**FIGURE 7:**
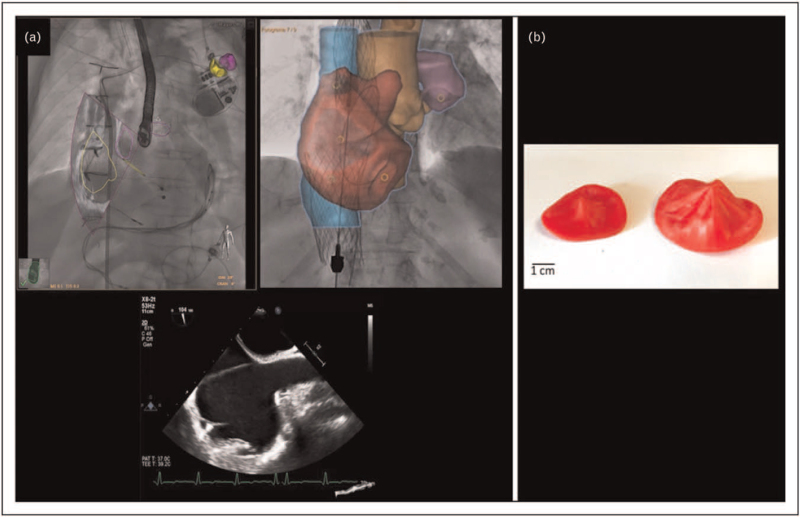
Example of fusion imaging during transcatheter procedure showing transesophageal echocardiographic bicaval view (a, bottom) superimposed on fluoroscopic images (a, top; courtesy of Prof. Jose L. Zamorano, University Hospital Ramon y Cajal, Madrid, Spain). Three-dimensional printing of a normal tricuspid valve (b, left) and a remodeled tricuspid valve (b, right) showing tricuspid annular dilatation and tethering of the leaflets.

The use of fluoroscopy is mandatory during percutaneous procedures to position wires and guiding catheters. However, fluoroscopy doesn’t allow landmark structure and TV visualization. Fusion imaging can overcome these limitations by the use of echocardiographic or MDCT images superimposed on fluoroscopic projections [76–79]. However, regardless of the technique used, 3D images are displayed on a 2D flat screen. The use of 3D printing [80] or virtual/extended reality might help interventional cardiologists to explore TV anatomy and function in all spatial directions and, possibly plan personalized treatment and guide transcatheter procedures [81] (Fig. 7).

## CONCLUSION

Currently, a multimodality imaging approach is necessary for the comprehensive assessment of the complex anatomy of the TV and right heart chambers. Accurate planning of TV intervention rely on several imaging modalities requiring advanced skills and multidisciplinary teamwork.

## Acknowledgements

*We would like to thank Prof. J.L. Zamorano (University Hospital Ramon y Cajal, Madrid, Spain) and Dr G. Muscogiuri (Centro Cardiologico Fondazione Monzino, IRCCS, Milan, Italy) for providing interventional fusion images and cardiac magnetic resonance 4D flow quantitation, respectively. We would like also to thank Tristan Slots (Pie Medical Imaging, The Netherlands) for the postprocessing of computed tomography images. Finally, we would like to thank Sergio Caravita, M.D., Ph.D.; Michele Tomaselli, M.D.; and Giorgio Oliverio, M.D., for their contribution to the review.*

### Financial support and sponsorship

*None.*

### Conflicts of interest

*L.P.B. and D.M. are on the speaker's bureau for GE Healthcare; L.P.B. has received honoraria from GE Healthcare, Philips Healthcare, Edward Lifesciences, Esaote S.p.A and Livanova S.p.A. V.V. is on the speaker's bureau for Philips Healthcare. The remaining authors have no conflicts of interest.*

## REFERENCES AND RECOMMENDED READING

Papers of particular interest, published within the annual period of review, have been highlighted as:

▪ of special interest
▪▪ of outstanding interest
